# *BMPR1B* mutation causes Pierre Robin sequence

**DOI:** 10.18632/oncotarget.16531

**Published:** 2017-03-23

**Authors:** Yongjia Yang, Jianying Yuan, Xu Yao, Rong Zhang, Hui Yang, Rui Zhao, Jihong Guo, Ke Jin, Haibo Mei, Yongqi Luo, Liu Zhao, Ming Tu, Yimin Zhu

**Affiliations:** ^1^ The Laboratory of Genetics and Metabolism, Hunan Children's Research Institute, Hunan Children's Hospital, University of South China, Changsha, China; ^2^ Institute of Emergency Medicine, People's Hospital of Hunan Province, Changsha, China; ^3^ BGI-Shenzhen, Shenzhen, China; ^4^ Division of Neonatology, Hunan Children's Hospital, University of South China, Changsha, China; ^5^ State Key Laboratory of Medical Genetics, Central South University, Changsha, China

**Keywords:** BMPR1B, Pierre Robin sequence, gene fusion, BMP signalling, cleft palate, Chromosome Section

## Abstract

**Background:**

We investigated a large family with Pierre Robin sequence (PRS).

**Aim of the study:**

This study aims to determine the genetic cause of PRS.

**Results:**

The reciprocal translocation t(4;6)(q22;p21) was identified to be segregated with PRS in a three-generation family. Whole-genome sequencing and Sanger sequencing successfully detected breakpoints in the intragenic regions of *BMRP1B* and *GRM4*. We hypothesized that PRS in this family was caused by (i) haploinsufficiency for *BMPR1B* or (ii) a gain of function mechanism mediated by the *BMPR1B*-*GRM4* fusion gene. In an unrelated family, we identified another *BMPR1B*-splicing mutation that co-segregated with PRS.

**Conclusion:**

We detected two *BMPR1B* mutations in two unrelated PRS families, suggesting that *BMPR1B* disruption is probably a cause of human PRS.

**Methods:**

GTG banding, comparative genomic hybridization, whole-genome sequencing, and Sanger sequencing were performed to identify the gene causing PRS.

## INTRODUCTION

Pierre Robin sequence (*PRS*, MIM: 261800), also known as *Pierre Robin syndrome*, is a condition characterized by micrognathia, cleft palate, and glossoptosis (leading to airway obstruction and eventually develops into chonechondrosternon) [1–3]. Most neonates with PRS have respiratory and feeding difficulties [1–3] (Supplementary Video 1 shows a typical video of a patient with PRS). PRS is also a subgroup of cleft lip and/or cleft palate, which are among the most common birth defects worldwide occurring at a rate of 1 to 2 per 1000 births [4]. PRS can be isolated or can be a part of several disorders, such as Stickler syndrome, velocardiofacial syndrome, fetal alcohol syndrome, and Treacher Collins syndrome [5, 6].

Several gene intervals of 2q24.1-33.3, 4q21-qter, 11q21-23.1, and 17q21-24.3 are associated with PRS [3]. Many studies have identified translocation breakpoints and mutations in the conserved and non-coding elements of SOX9 in patients with PRS; results illustrated that SOX9 disruptions are involved in PRS [7, 8].

In this study, we performed GTG banding, comparative genomic hybridization, whole-genome sequencing, and Sanger sequencing in blood samples obtained from two PRS families and controls.

## RESULTS

### GTG banding and array CGH

**Figure 1:**
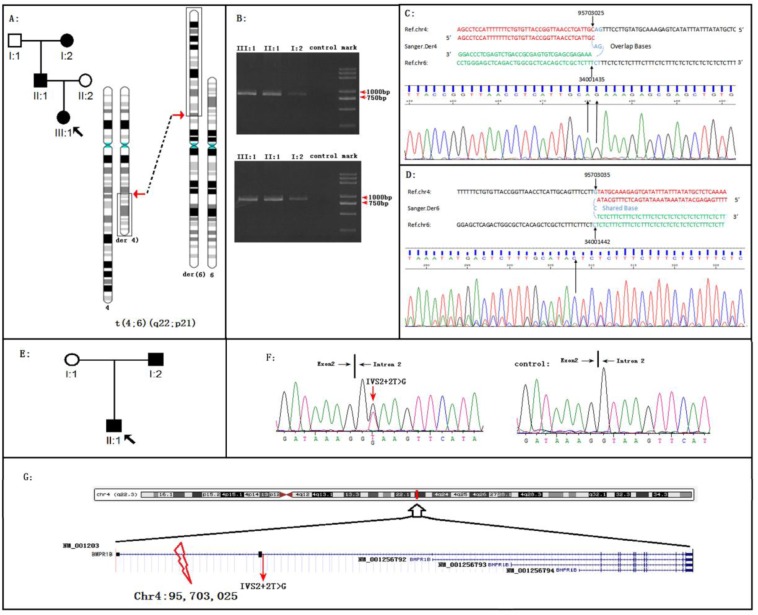
*BMPR1B* disruptions cause Pierre Robin Syndrome (PRS) in two unrelated families **A**. The PRS family 01 with the reciprocal translocation t(4;6)(q22;p22). **B**. Migration of amplified junction fragments on agarose gels. After whole genome sequencing, the junction fragments were amplified by the polymerase chain reaction (PCR). The PCR products of Der4 (upper panel) and Der6 (down panel) migrated in the 0.8% agarose gel. Two pairs of primers for Der4 (6-4F: AAGTCCCAGGGCATGAGGTATC and 6-4R: GCTTTGGTATCATATTAACCATTGC) and Der6 (4-6F: TGGTTGAATATAGGAGTCTGGAGT and 4-6R: AGGTCAATGGAGATAGTACAACCT) were used for PCR. The length of both predicted fragments is 800 bp. **C**. On the proband of family 01, Sanger sequencing of the fragment derived from DER 4 defined the breakpoints in the base-pair resolution: 4q22.3:95703025 fused to 6p21:34001435; AG was the shared sequence. **D**. On the proband of family 01, Sanger sequencing of the fragment derived from DER 6 defined the breakpoints in the base-pair resolution: 4q22.3:95703035 fused to 6p21.31:34001442; C was the shared sequence. **E**. The PRS family 02. **F**. Sequence chromatograms of the BMPR1B splicing mutation IVS2+2T>G. The mutation was occurred on I:2 and II:1 (left) but not on I:1 (right) in the family. **G**. The schematic of positions of two BMPR1B disruptions. Note: the human BMPR1B include four different transcripts, all two BMPR1B disruptions were occurred on the BMPR1B NM_001203 transcript.

**Figure 2:**
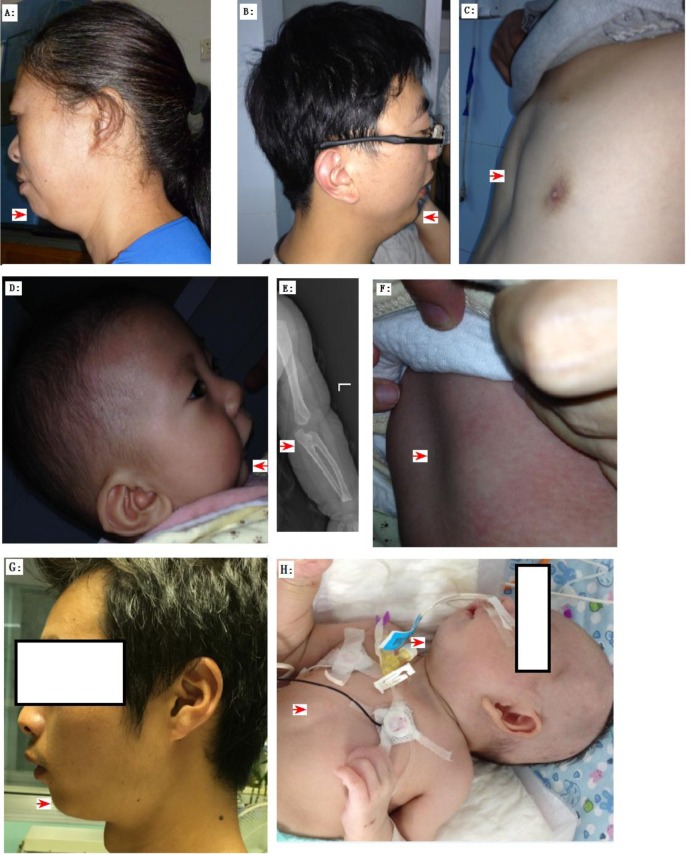
The clinic phenotypes of Family 01 A.-F. and Family 02 (G-H) with Pierre Robin Syndrome (PRS) Family 01: **A**. The PRS member I: 2 exhibited micrognathia and ‘a bird like head’. **B**. The PRS member II: 1 exhibited micrognathia and ‘a bird like head’. **C**. The PRS member II: 1 exhibited pectus excavatum. **D**.-**F**. The PRS member III: 1 exhibited micrognathia, radioulnar synostosis (radioulnar synostosis is a rare phenotype of PRS ref34), and pectus excavatum. Family 02: **G**. The PRS member I: 2 exhibited micrognathia and ‘a bird like head’. **H**. The PRS member II: 1 exhibited micrognathia and pectus excavatum.

**Figure 3:**
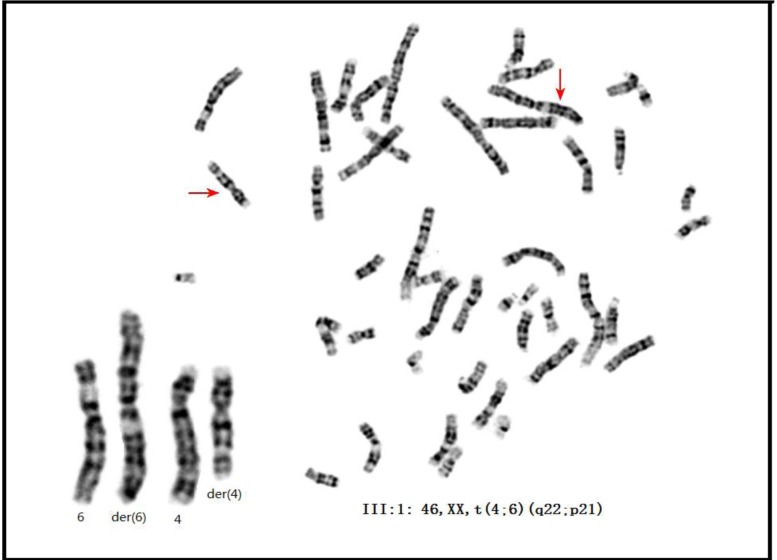
The karyotype of the proband of family 01 GTG-banding on III:1 detected the karyotype of 46, XX, t(4;6)(q22;p21). Note: the karyotypes of the I:2 and II:1 were presented in Figure S4.

We investigated and performed GTG banding on two PRS-affected families (Figure 1, Figure 2, and Table 1). All the affected members in family 01 carried the t(4;6)(q22;p21) reciprocal translocation (Figure 3, Supplementary Figure S1), whereas the unaffected members in family 01 carried none (data not shown). In family 02, GTG banding (550-800 band level) was not able to detect any chromosomal abnormality (data not shown). Comparative genomic hybridization (CGH) was also performed on all three PRS-affected members in family 01 because apparent balanced translocations probably produce copy number variations (CNVs). No pathogenic CNVs were detected near 4q22, 6p21, or any other location in the PRS-affected family 01 (data not shown). CGH was also performed on the proband (II:1) in family 02 (and other 7 PRS sporadic cases, Supplementary Table S2). No pathogenic CNVs were detected (particularly in 4q22 and 6p21 areas, data not shown).

**Table 1:** Clinical data of patients in two families with Pierre Robin Syndrome

individuals	F01-I:2	F01-II:1	F01-III:1	F02-I:2	F02-II:1
sex	female	male	female	male	male
height	158 cm	171 cm	65.3 cm	165 cm	47cm
birth weight (kg)	2.3 kg(−2SD)	3.1 kg	2.15 kg (−3SD)	2.4 kg (−2SD)	2.6 kg(−1SD)
age (years)	52	31	0.6	29	0.1
micrognathia	(+)	(+)	(+)	(+)	(+)
cleft palate	(+)	(−) high arch palate	(+)	(−)	(+)
glossoptosis	(+)	(+)	(+)	(+)	(+)
chonechondrosternon	(+)	(+)	(+)	(−)	(+)
radioulnar synostosis	(+)	(−)	(−)	(−)	(−)

**Figure 4:**
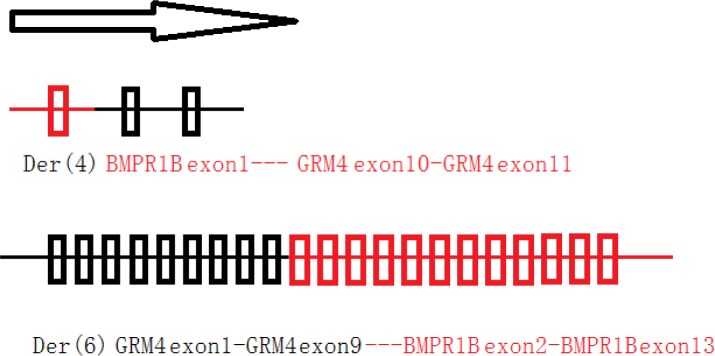
Two deduced fusion-genes in PRS family 01

### Whole genome sequencing and Sanger sequencing (WGLCS)

In family 01, WGLCS was successfully conducted on all three affected members. Both der (4) and der (6) breakpoints were successfully detected and confirmed immediately by Sanger sequencing (Figure 1b-1d). We also detected the same breakpoints on all three affected members, suggesting that the 4q22 and 6p21 translocations were steadily transmitted in family 01 with PRS (Figure 1b-1d, Supplementary Figure S2, and Supplementary Figure S3). At 4q22, the breakpoint occurred on Chr4: 95,703,025 (Figure 1c, Supplementary Figure S2, and Supplementary Figure S3). Thus, the BMP receptor type 1B (*BMPR1B*) gene was truncated by fusing the first intron of *BMPR1B* (NM_001203) to 6p21 at Chr6: 34,001,435 in the eighth intron of *GRM4* (Figure 1g and Supplementary Figure S4). We hypothesized that two fusion genes were produced (Figure 4). In family 02 (Figure 1e), Sanger sequencing on the coding regions, namely, exon-intron boundaries of *BMPR1B* and *GRM4*, was successfully carried out. After filtering common variants present in 1000 Genomes (www.1000genomes.org), we detected a splicing mutation (IVS2+2T > G) of *BMPR1B* segregating with PRS in family 02 (Figure 1f). This *BMPR1B*-IVS2+2T > G mutation was absent in 496 regions, ethnic-matched controls, and other public databases (dnSNP, YH database, and 800 in-house exome data).

### Blood cDNA analysis

We identified the *BMPR1B*-*GRM4* (or vice versa) fusion transcript in family 01 and find the altered *BMPR1B* transcripts in family 02. The PCR primer was successfully designed (Supplementary Table S1). In the analysis of cDNA template from the peripheral lymphocytes of a healthy control, both the amplified *BMPR1B* and *GRM4* transcripts were not detected on polyacrylamide gel. Hence, *BMPR1B* and *GRM4* were not expressed in the blood or were expressed in the blood at very low levels. *BMPR1B* was expressed in cartilaginous tissues. However, other informative tissues from patients belong to family 01 and 02 were unavailable.

## DISCUSSION

This research investigated two PRS-affected families with *BMPR1B* disruptions. In family 01 with reciprocal translocation, several advanced genetic techniques successfully and precisely detected the translocation breakpoints. The translocation segregated steadily with PRS through three generations in family 01. At 4q22, the breakpoint was between positions Chr4: 95, 703, 025 and Chr4: 95, 703, 028. This breakpoint interrupted the first intron of the *BMPR1B* gene (NM_001203) (Figure 1g). At 6p21, the breakpoint was between positions Chr6: 34, 001, 435 and Chr6: 34, 001, 436. The 6p21 breakpoint (Supplementary Figure S4) interrupted the eighth intron of *GRM4* (*GRM4*, NM_001282847), encoding an excitatory neurotransmitter, namely, the glutamate metabotropic receptor 4. The diseases associated with *GRM4* include epilepsy and schizophrenia [11]. In PRS-affected family 02, Sanger sequencing on the coding regions and exon-intron boundaries of *BMPR1B* or *GRM4* detected a mutation (a splicing mutation IVS2+2T>G) on *BMPR1B* but none on *GRM4*.

Bone and joint morphogenesis must be precisely orchestrated by the bone morphogenic protein (BMP) signaling pathway [12]. This pathway includes BMP ligands, BMP receptors, and BMP antagonists. BMP ligand mutations cause human CL/P and BMP antagonist variants associated with human CL/P [13–22].

As a BMP receptor, the human *BMPR1B* gene is located in 4q22. This gene induces the formation of bones and joints [23, 24] and is essential for precise BMP signaling in bone and joint morphogenesis [23, 24]. The 4q21-qter is a previously defined CL/P (or PRS) locus. At least three studies suggested the presence of CL/P disease gene [25–27], and two studies indicated the presence of PRS disease gene [2, 28] located in the 4q21-qter interval.

Previous studies reported the presence of *BMPR1B* mutations in patients with human joint fusion and limb deformities, including brachydactyly type A2 [29], brachydactyly type C/symphalangism-like phenotype [30], and du Pan acromesomelic dysplasia [31]. Consistently, the proband of family 01 exhibited the joint fusion phenotype, namely, radio-ulnar synostosis (RUS) (Figure 2e). RUS, a congenital joint disease [32], can either be sporadic or transmitted with the inheritance of autosomal dominant [33]. RUS is a rare phenotype of PRS [34]. In this study, we investigated another RUS family (family 03, Supplementary Figure S5a) with two members having RUS and phalangeal dysplasia or isolated RUS (Supplementary Figure S6), but without PRS phenotypes. In this family, Sanger sequencing on *BMPR1B* detected a missense variant that cosegregated with the disease (c.1024A > G/p.Lys342Glu) (Supplementary Figure S5b). This variant was absent in 496 region- and ethnic-matched controls and in public databases, including 1000 Genomes and 800 in-house exomes. Lysine was substituted by glutamic acid in 342 residues located in a highly conserved region of *BMPR1B* (Supplementary Figure S7), and three functional predicting software (PolyPhen, Sift, and MutationTaster); predict this variant can cause a disease. However, the pathogenicity of this variant remains unclear because of the following: incomplete penetrance of the family investigated and the variant was hit two times in the ExAc database. This missense mutation must be subjected to further functional analysis.

The human BMPR1B protein possesses four different transcripts (Figure 1g), which are NM_001256794, NM_001256792, NM_001203, and NM_001256793. NM_001203 is the longest transcript (Figure 1g) and has a long non-protein-coding 5′ UTR, which is encoded by exons 1-4 (Figure 1g). In this study, both *BMPR1B* mutations of family 01 and family 02 are located on the NM_001203 specific region (Figure 1g, the exon 1-3 region).

In summary, we identified two *BMPR1B* disruptions in two unrelated PRS-affected families. We also observed that both PRS disruptions occurred on the NM_001203 transcript of *BMPR1B*, and mutation occurring on other transcripts/locations did not exhibit CL/P phenotypes. These observations suggest that (i) the NM_001203 transcript may play important roles for human mandible and palate morphogenesis, and (ii) different transcripts of *BMPR1B* may play spatial-specific roles in human skeletal morphogenesis. This study is the first to report that BMP receptor disruption can cause human CL/P spectrum disorders.

## MATERIALS AND METHODS

### Study subjects

The project was approved by the ethics committee of the Hunan Children's Hospital, Changsha City, China. Before this study, all subjects and child guardians signed a written informed consent. This study involved two PRS families (Table 1 presents the detailed clinical data for 5 PRS members of these two families), including family 01 (Figure 1a and Figure 2a-2f) and family 02 (Figure 1g and Figure 2g-2h), and 496 ethnic- and region-matched controls. These controls have neither diagnostic features nor family history of PRS. For each subject, genomic DNA was extracted from peripheral blood (1-3 mL in heparin sodium tubes) through the phenol/trichloromethane method as described by the standard protocol.

### Chromosome GTG banding and array CGH

The Giemsa banding (400-550 bands lever) was conducted on all members of these two PRS families. High resolution Giemsa banding was also carried out in these two PRS families in accordance with the standard laboratory protocol. The Agilent 4×180 K commercial arrays (Agilent Technologies, Santa Clara, CA, USA) were used to detect CNV on four affected members of PRS families (the I:2, II:1 and III:1 in family 01, Figure 1a; the II:1 of family 02, Figure 1g ). The experimental and analytical pipelines of the array conformed to the standard protocol as previously described [9].

### Whole genome sequencing

We used whole genome low-coverage sequencing (WGLCS) to detect the breakpoints of family 01. The detailed method for WGLCS is available in a previously published paper [9]. In brief, 500 ng genomic DNA for each individual was subjected to the following steps: DNA sheared into small fragments, end-repair, “A”-overhanging and adapter-ligation, fragment (about 625 bp long) selection in agarose gel electrophoresis, PCR with multiplex primers, and fragment selection in agarose gel. The Agilent Bio-analyzer DNA 1000 kit (Agilent Technologies) was used to determine the size distribution of the library. The library concentration was measured by quantitative PCR, and libraries with index tags were sequenced by Illumina HiSeq 2000 platform. Data analysis was performed through the following steps: (1) removal of the reads of adaptors and low-quality reads; (2) alignment of the reads to hg19 or GRCh37.1; and (3) translocation discovery, chimeric read-pair inputs for T-CHR (an in-house software [10]), analysis of clustering by locations, removal of PCR duplicates, and four steps of filtration [10].

### Sanger sequencing

Sanger sequencing was performed on the basis of the following experiments: (i) the detection of breakpoint regions for family 01, and (ii) the mutation scanning of the exon and exon/intron junctions of the BMPR1B (NM_001203) and the GRM4 (NM_001282847) gene in family 02. The primers were designed using the software Primer3 (http://frodo.wi.mit.edu) and were synthesized by local biotech company. Primer sequences and the PCR conditions are available on request. The BigDye 3.1 Mix Kit (Applied Biosystems, Foster City, CA) was used for all seq reactions. Dye-labeled PCR products were purified in 70% alcohol and electrophoresed on an ABI 3500 genetic analyzer (Applied Biosystems, Foster City, CA). All the sequencing data were analyzed using the SEQMAN software (DNA Star Package, WI, USA).

### Blood RNA extraction and RT-PCR

Lymphocytes were separated from 2 mL heparin-anticoagulated venous blood using Lynmphocyte separation medium(Solarbio, Shanghai, China). Total RNA was extracted from lymphocytes using RNAiso Plus Kit (TaKaRa, Dalian, China). cDNA was synthesized from 1ug of RNA using the PrimeScript™ II 1st Strand cDNA Synthesis Kit(TaKaRa, Dalian, China). PCR reactions were performed on a 2700 PCR System (Applied Biosystems).

For the detection of fusion-genes (in family 01) or to value the effect of the splice site variant (in family 02), PCR primers were synthesized (Supplementary Table 1).

## SUPPLEMENTARY MATERIALS FIGURES AND TABLES





## References

[1] Amaddeo A, Abadie V, Soupre V, Chalouhi C, Bolanos MF, Pierrot S, Frapin A, Lapillone A, Ramirez A, Picard A, Fauroux B (2015). Management of upper airway obstruction by non-invasive CPAP in neonates with Pierre Robin sequence. European Respiratory Journal.

[2] Wright M, Mehendale F, Urquhart D (2014). Utility of sleep studies in Pierre Robin sequence: A 10-year study. European Respiratory Journal.

[3] Jakobsen LP, Knudsen MA, Lespinasse J, García Ayuso C, Ramos C, Fryns JP, Bugge M, Tommerup N (2006). The genetic basis of the Pierre Robin Sequence. Cleft Palate Craniofac J.

[4] Watkins SE, Meyer RE, Strauss RP, Aylsworth AS (2014). Classification, epidemiology, and genetics of orofacial clefts. Clin Plast Surg.

[5] van den Elzen AP, Semmekrot BA, Bongers EM, Huygen PL, Marres HA, van den Elzen AP, Semmekrot BA, Bongers EM (2001). Diagnosis and treatment of the Pierre Robin sequence: results of a retrospective clinical study and review of the literature. Eur J Pediatr.

[6] Shprintzen RJ (1992). The implications of the diagnosis of Robin sequence. Cleft Palate Craniofac J.

[7] Jakobsen LP, Ullmann R, Christensen SB, Jensen KE, Mølsted K, Henriksen KF, Hansen C, Knudsen MA, Larsen LA, Tommerup N, Pierre Tümer Z (2007). Robin sequence may be caused by dysregulation of SOX9 and KCNJ2. Journal of Medical Genetics.

[8] Benko S, Fantes JA, Amiel J, Kleinjan DJ, Thomas S, Ramsay J, Jamshidi N, Essafi A, Heaney S, Gordon CT, McBride D, Golzio C, Fisher M (2009). Highly conserved non-coding elements on either side of SOX9 associated with Pierre Robin sequence. Nat Genet.

[9] Yang Y, Yang C, Zhu Y, Chen H, Zhao R, He X, Tao L, Wang P, Zhou L, Zhao L, Tu M, Dong Z, Chen H (2014). Intragenic and extragenic disruptions of FOXL2 mapped by whole genome low-coverage sequencing in two BPES families with chromosome reciprocal translocation. Genomics.

[10] Dong Z, Jiang L, Yang C, Hu H, Wang X, Chen H, Choy KW, Hu H, Dong Y, Hu B, Xu J, Long Y, Cao S (2014). A robust approach for blind detection of balanced chromosomal rearrangements with whole-genome low-coverage sequencing. Hum Mutat.

[11] Parihar R, Mishra R, Singh SK, Jayalakshmi S, Mehndiratta MM, Ganesh S (2014). Association of the GRM4 gene variants with juvenile myoclonic epilepsy in an Indian population. J Genet.

[12] Lehmann K, Seemann P, Stricker S, Sammar M, Meyer B, Süring K, Majewski F, Tinschert S, Grzeschik KH, Müller D, Knaus P, Nürnberg P, Mundlos S (2003). Mutations in bone morphogenetic protein receptor 1B cause brachydactyly type A2. Proc Natl Acad Sci USA.

[13] Sahoo T, Theisen A, Sanchez-Lara PA, Marble M, Schweitzer DN, Torchia BS, Lamb AN, Bejjani BA, Shaffer LG, Lacassie Y (2011). Microdeletion 20p12.3 involving BMP2 contributes to syndromic forms of cleft palate. Am J Med Genet A.

[14] Williams ES, Uhas KA, Bunke BP, Garber KB, Martin CL (2012). Cleft palate in a multigenerational family with a microdeletion of 20p12.3 involving BMP2. Am J Med Genet A.

[15] Suazo J, Tapia JC, Santos JL, Castro VG, Colombo A, Blanco R (2011). Risk variants in BMP4 promoters for nonsyndromic cleft lip/palate in a Chilean population. BMC Med Genet.

[16] Suzuki S, Marazita ML, Cooper ME, Miwa N, Hing A, Jugessur A, Natsume N, Shimozato K, Ohbayashi N, Suzuki Y, Niimi T, Minami K, Yamamoto M Mutations in BMP4 are associated with subepithelial, microform, and overt cleft lip. Am J Hum Genet.

[17] Kouskoura T, Kozlova A, Alexiou M, Blumer S, Zouvelou V, Katsaros C, Chiquet M, Mitsiadis TA, Graf D (2013). The etiology of cleft palate formation in BMP7-deficient mice. PLoS One.

[18] Yu Q, He S, Zeng N, Ma J, Zhang B, Shi B, Jia Z (2015). BMP7 Gene involved in nonsyndromic orofacial clefts in Western Han Chinese. Med Oral Patol Oral Cir Bucal.

[19] Leslie EJ, Taub MA, Liu H, Steinberg KM, Koboldt DC, Zhang Q, Carlson JC, Hetmanski JB, Wang H, Larson DE, Fulton RS, Kousa YA, Fakhouri WD (2015). Identification of functional variants for cleft lip with or without cleft palate in or near PAX7, FGFR2, and NOG by targeted sequencing of GWAS loci. Am J Hum Genet.

[20] Song T, Shi J, Guo Q, Lv K, Jiao X, Hu T, Sun X, Fu S (2015). Association between NOGGIN and SPRY2 polymorphisms and nonsyndromic cleft lip with or without cleft palate. Am J Med Genet A.

[21] Matsui M, Klingensmith J (2014). Multiple tissue-specific requirements for the BMP antagonist Noggin in development of the mammalian craniofacial skeleton. Dev Biol.

[22] Anderson RM, Lawrence AR, Stottmann RW, Bachiller D, Klingensmith J (2002). Chordin and noggin promote organizing centers of forebrain development in the mouse. Development.

[23] Chen D, Zhao M, Mundy GR (2004). Bone morphogenetic proteins. Growth Factors.

[24] Gazzerro E, Canalis E (2006). Bone morphogenetic proteins and their antagonists. Rev Endocr Metab Disord.

[25] Schultz RE, Cooper ME, Daack-Hirsch S, Shi M, Nepomucena B, Graf KA, O'Brien EK, O'Brien SE, Marazita ML, Murray JC (2004). Targeted scan of fifteen regions for nonsyndromic cleft lip and palate in Filipino families. Am J Med Genet A.

[26] Brewer C, Holloway S, Zawalnyski P, Schinzel A, FitzPatrick D (1998). A chromosomal deletion map of human malformations. Am J Hum Genet.

[27] Wyszynski DF, Albacha-Hejazi H, Aldirani M, Hammod M, Shkair H, Karam A, Alashkar J, Holmes TN, Pugh EW, Doheny KF, McIntosh I, Beaty TH, Bailey-Wilson JE (2003). A genome-wide scan for loci predisposing to non-syndromic cleft lip with or without cleft palate in two large Syrian families. Am J Med Genet A.

[28] Bhoj E, Halbach S, McDonald-McGinn D, Tan C, Lande R, Waggoner D, Zackai E (2013). Expanding the spectrum of microdeletion 4q21 syndrome: a partial phenotype with incomplete deletion of the minimal critical region and a new association with cleft palate and Pierre Robin sequence. Am J Med Genet A.

[29] Lehmann K, Seemann P, Stricker S, Sammar M, Meyer B, Süring K, Majewski F, Tinschert S, Grzeschik KH, Müller D, Knaus P, Nürnberg P, Mundlos S (2003). Mutations in bone morphogenetic protein receptor 1B cause brachydactyly type A2. Proc Natl Acad Sci U S A.

[30] Lehmann K, Seemann P, Boergermann J, Morin G, Reif S, Knaus P, Mundlos S (2006). A novel R486Q mutation in BMPR1B resulting in either a brachydactyly type C/symphalangism-like phenotype or brachydactyly type A2. Eur J Hum Genet.

[31] Stange K, Désir J, Kakar N, Mueller TD, Budde BS, Gordon CT, Horn D, Seemann P, Borck G (2015). A hypomorphic BMPR1B mutation causes du Pan acromesomelic dysplasia. Orphanet J Rare Dis.

[32] Zhu Y, Jin K, Mei H, Li L, Liu Z, Yang Y, Tang J, He X, Zhao R, He X (2012). A family with radio-ulnar synostosis, scoliosis, and thick vermilion of lips: a novel syndrome or variant of Giuffrè-Tsukahara syndrome?. Am J Med Genet A.

[33] Rizzo R, Pavone V, Corsello G, Sorge G, Neri G, Opitz JM (1997). Autosomal dominant and sporadic radio-ulnar synostosis. Am J Med Genet.

[34] Ozkan KU, Coban YK, Uzel M, Ergun M, Oksuz H (2006). Pierre Robin sequence with esophageal atresia and congenital radioulnar synostosis. Cleft Palate Craniofac J.

