# Supplementary material for: BMPR1B mutation causes Pierre Robin sequence
Source: Oncotarget. 2017 Mar 23;8(16):25864–71. doi: 10.18632/oncotarget.16531 (PMC5432222; doi:10.18632/oncotarget.16531)
Supplement: Supplementary file 1 [file oncotarget-08-25864-s001.pdf]

# *BMPR1B* mutation causes Pierre Robin sequence

## Supplementary Material

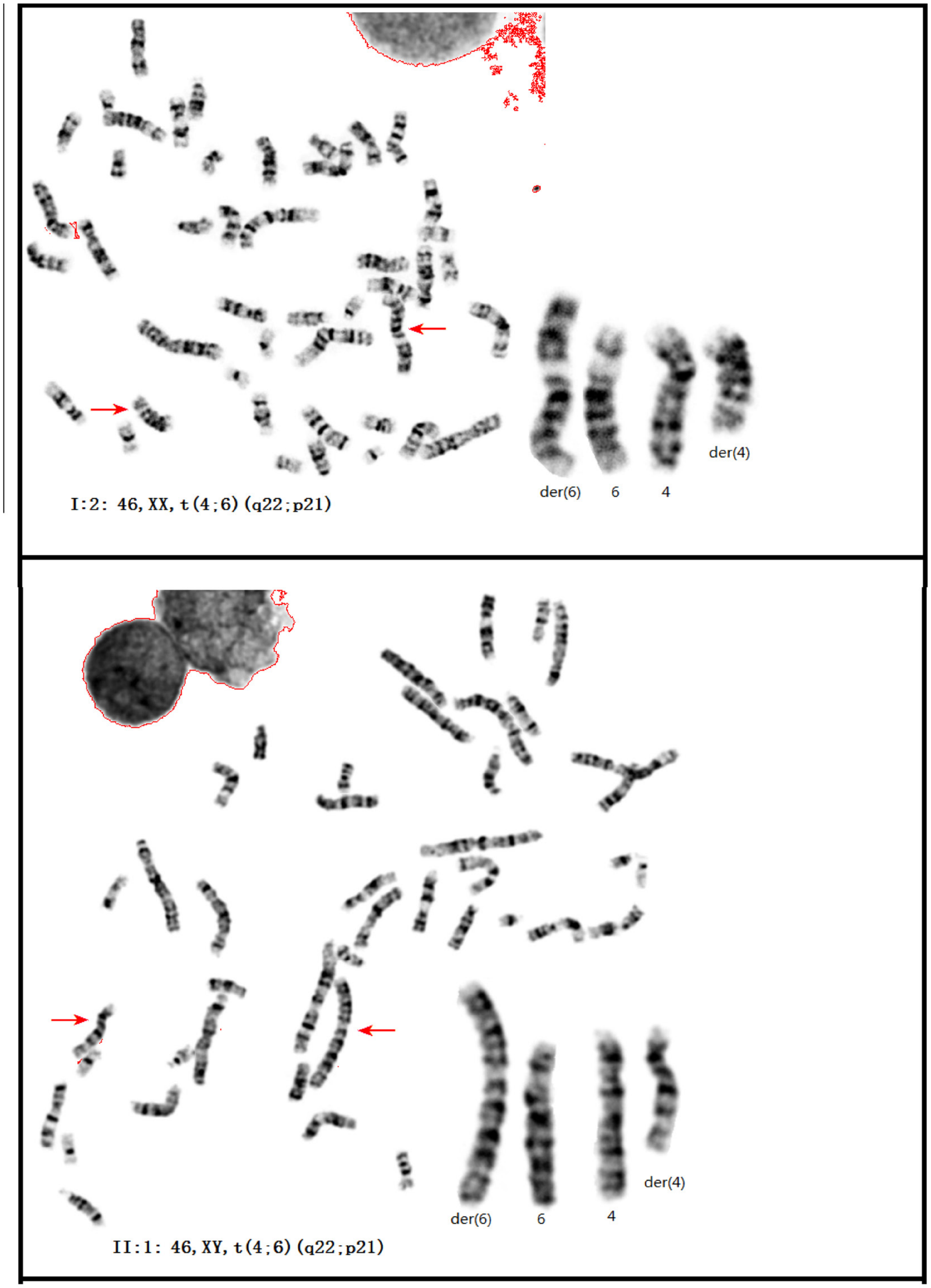


**Supplementary Figure 1: The karyotypes of the family 01.** GTG-banding carried on I:2 and II:1 detected the karyotype of 46, XX, t(4;6)(q22;p21) and 46, XY, t(4;6)(q22;p21), respectively.

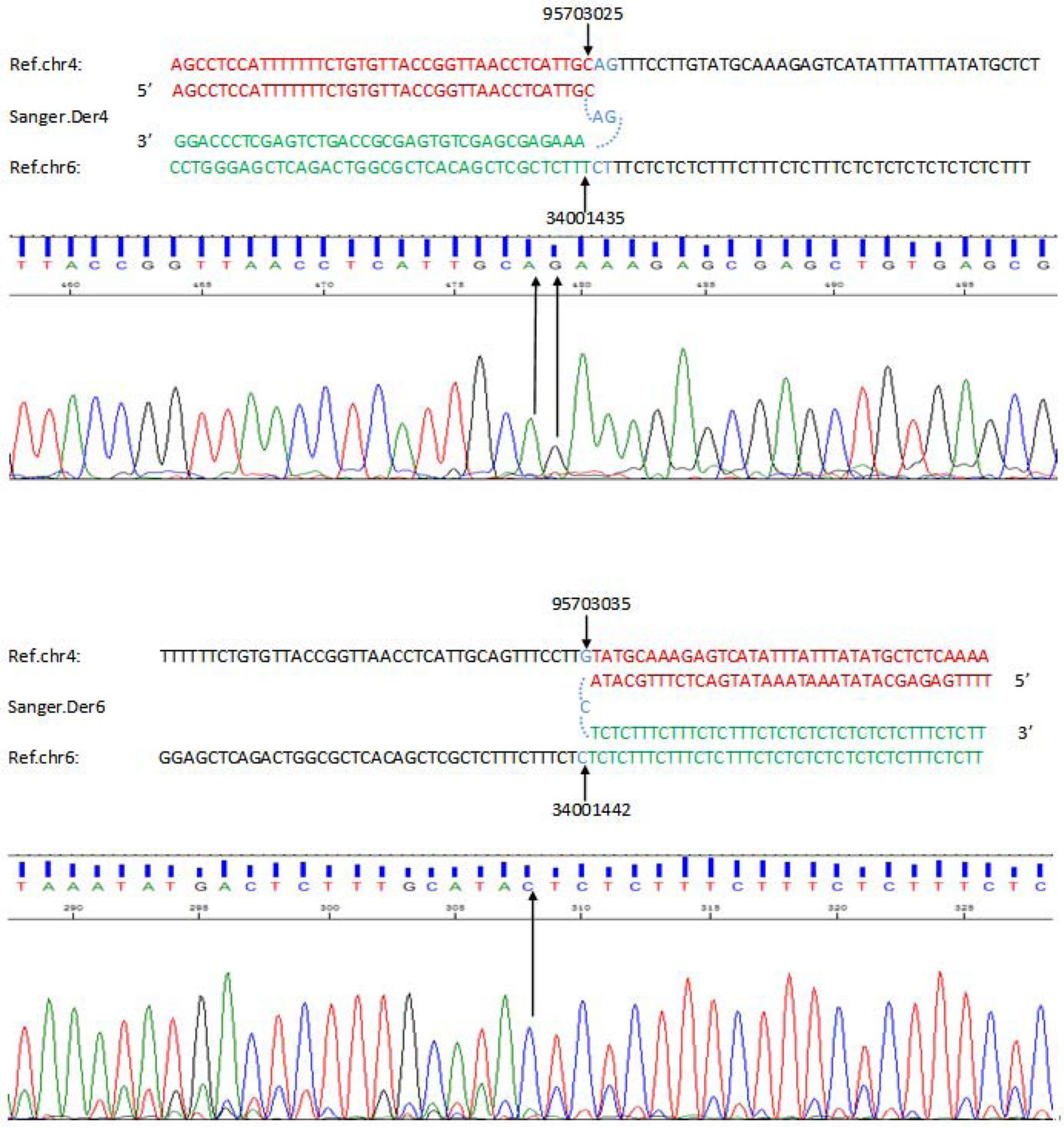


**Supplementary Figure 2: On I:2 of family 01, Sanger sequencing of the fragment derived from DER 4 and DER 6 allowed defining breakpoints at base pair resolution.** The 4q22.3: 95703025 fused to the 6p21.31: 34001435. The AG was sharing sequence. The 4q22.3: 95703035 fused to the 6p21.31: 34001442. The C was sharing sequence.

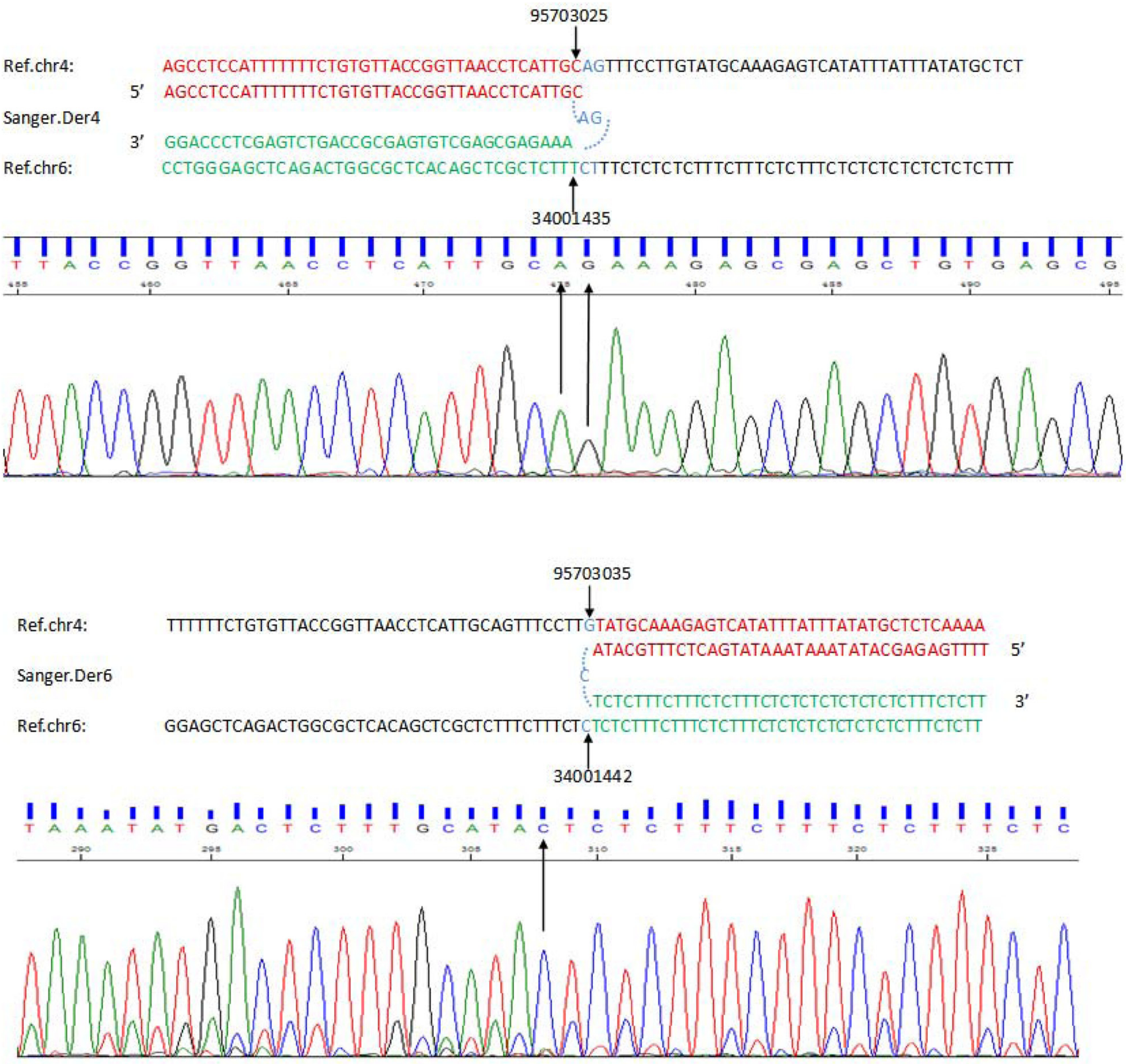


**Supplementary Figure 3: On II:1 of family 01, Sanger sequencing of the fragment derived from DER 4 and DER 6 allowed defining breakpoints at base pair resolution.** The 4q22.3: 95703025 fused to the 6p21.31: 34001435. The AG was sharing sequence. The 4q22.3: 95703035 fused to the 6p21.31: 34001442. The C was sharing sequence.

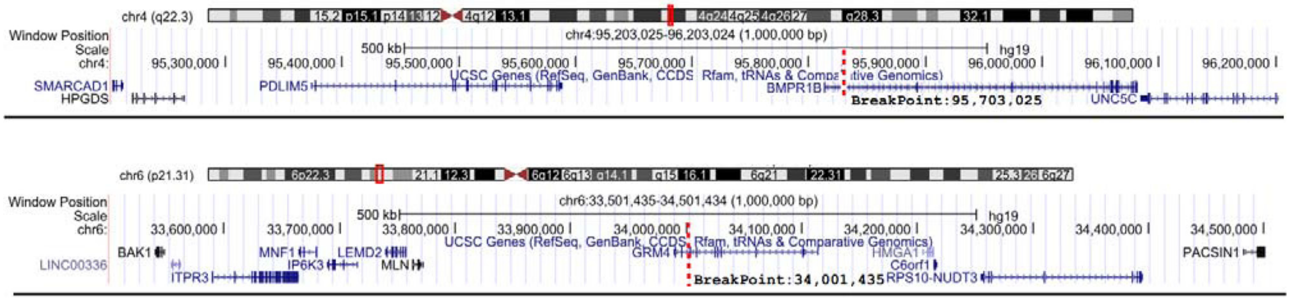


**Supplementary Figure 4: The overview of the 4q22 and 6p21 breakpoints in the PRS family 01.**

[illegible]

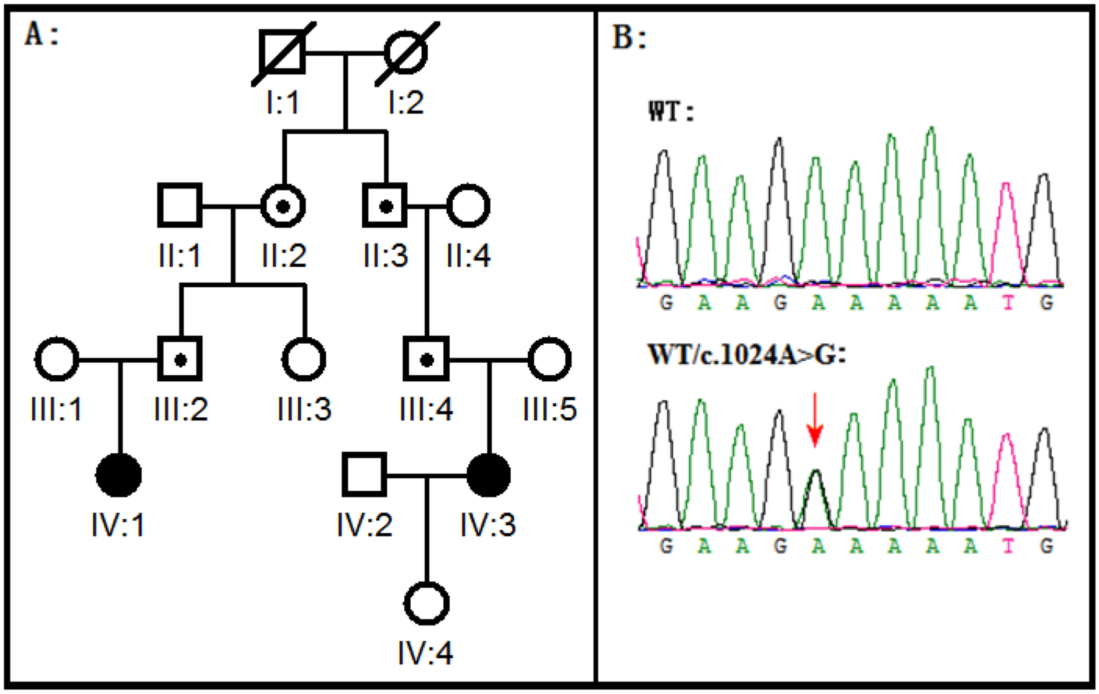


**Supplementary Figure 5: The family 03 and the BMPR1B variant c.1024A>G.** (a) The pedigree of family 03; (b) Sequence chromatograms of the BMPR1B missense variant (c.1024A>G/ p.Lys342Glu). This variant (upper) was detected on the family cases II:2, II:3, III:2, III:4, IV:1 and IV:3 but not on II:1, II:4, III:1, III:3, III:5, IV:2 and IV:4.

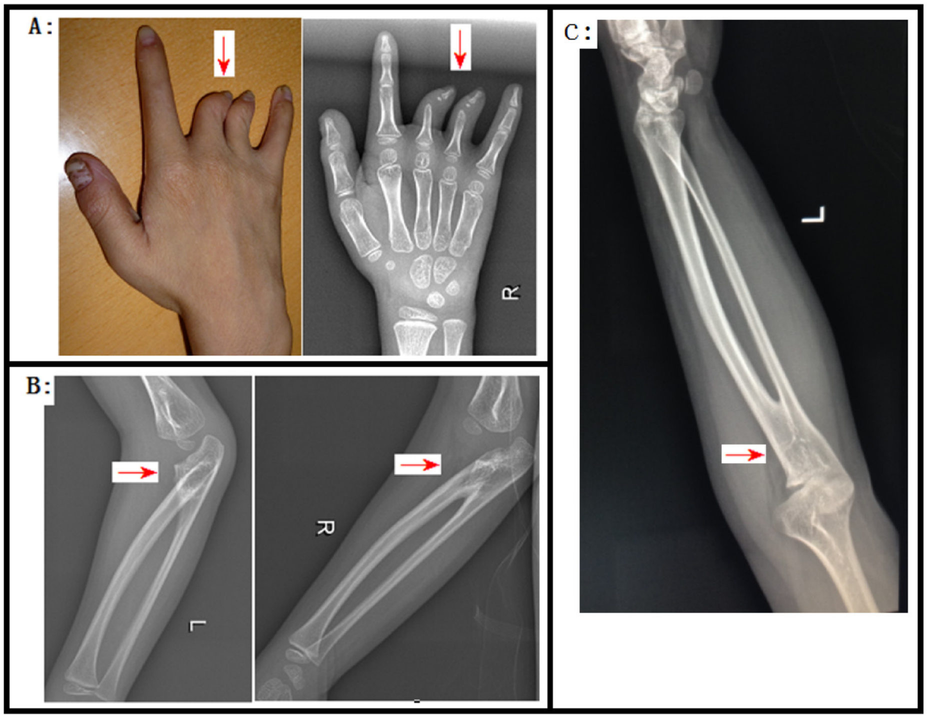


**Supplementary Figure 6: The phenoypes of the family 03 with BMPR1B c.1024A>G.** The IV:1 exhibited bilateral radioulnar synostosis (A) and the unilateral phalangeal dysplasia (b). The IV:3 exhibit unilateral radioulnar synostosis.

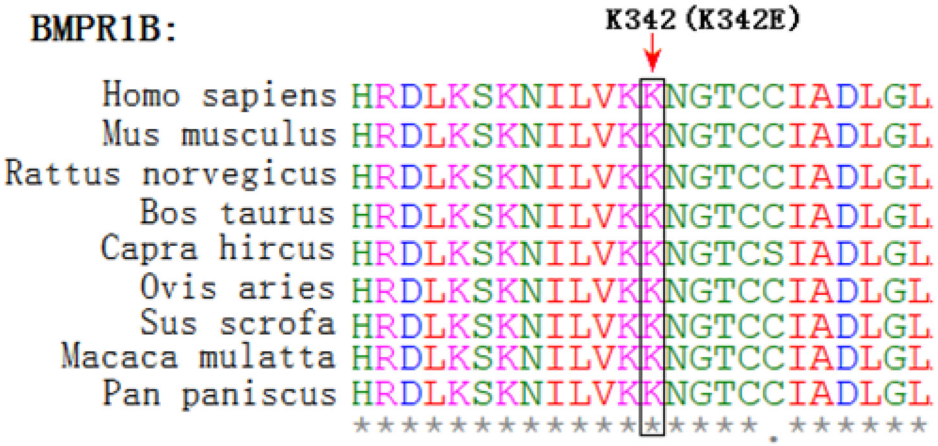


**Supplementary Figure 7: A multiple alignment of the amino acid sequence of Homo sapiens with the orthologous proteins Mus musculus, Rattus norvegicus, Bos Taurus, Capra hircus, Ovis aries, Sus scrofa, Macaca mulatta, and Pan paniscus for protein BMPR1B.**

**Supplementary Table 1:** Primer for cDNA amplification:

| Primers for the detection of splicing effects in family 02 | | |
|---|---|---|
| Name | primer sequence | size |
| bmpr1b-13f | CCGCGGGTTCAGACTTCT | 220bp |
| bmpr1b-13r | GGACAATGGTGGTGGCATTT | |
| bmpr1b-24f | AGGAAGATCATTTCATGCCTTGT | 233bp |
| bmpr1b-24r | TGGTGGCATTTACAACGCAA | |
| bmpr1b-25f | AGGAAGATCATTTCATGCCTTGT | 244bp |
| bmpr1b-25r | CAGAAGTGACCACAGGCAAC | |
| Primers for the detection of fusion-genes in family 01 | | |
| der4-1f | AGACCGCGGCGCTGAGGA | 391bp |
| der4-2f | CGCTGAGGACGCGGGAGC | |
| der4-3f | CTGAGGACGCGGGAGCCG | 392bp |
| der4-1r | TGGACTCGCTAGATTGCATGGT | |
| der4-2r | TGCTCAGCTCCATGGACTCG | 491bp |
| der4-3r | TGAACTTGTTGGACATGGTGGC | |
| der6-1f | GACCAGCGGACACTCGAC | 490bp |
| der6-1r | GTCATCCTCTTCTATCATCGTGA | |
| der6-2f | ACTCGGTGGTGGACTTCC | 255bp |
| der6-2r | GTGAAACAATATCCGTCTGTGC | |
| der6-3f | GGCATCTGTGTGTGGTTTGT | 330bp |
| der6-3r | TCCTCTTCTATCATCGTGAAACA | |
| der6-4f | GTGTGGTTTGTGGTGGACC | 221bp |
| der6-4r | TGAAAATCTGAGCCTTCTAGTCC | |
| der6-5f | CAGGACCAGCGGACACTC | 380bp |
| der6-5r | TCATCGTGAAACAATATCCGTCT | |

**Supplementary Table 2:** The clinic data of seven sporadic patients with Pierre Robin Syndrome.

| cases | sex | birth weight | age | karyotype | phenotype |
|---|---|---|---|---|---|
| m1666 | male | 2800g | 12D | 46,XY | pierre robbin syndrome |
| m1656 | female | 2050g | 3Y | 45,X[15]/46,X,r(X)[3] | pierre robbin syndrome,short stature |
| m1634 | male | 3100g | 6D | 46,XY | pierre robbin syndrome |
| m1596 | male | 1900g | 1M | 46,XY | pierre robbin syndrome |
| m1591 | female | 2200g | 3D | 46,XX | pierre robbin syndrome, radio ulnar synostosis and aricular anormaly |
| m1534 | male | 3250g | 4D | 46,XY | pierre robbin syndrome |
| m1529 | male | 2800g | 1Y2M | 46,XY | pierre robbin syndrome |
